# Mucosal-Associated Invariant T Cells Improve Nonalcoholic Fatty Liver Disease Through Regulating Macrophage Polarization

**DOI:** 10.3389/fimmu.2018.01994

**Published:** 2018-09-04

**Authors:** Yanmei Li, Bingyuan Huang, Xiang Jiang, Weihua Chen, Jun Zhang, Yiran Wei, Yong Chen, Min Lian, Zhaolian Bian, Qi Miao, Yanshen Peng, Jingyuan Fang, Qixia Wang, Ruqi Tang, M. Eric Gershwin, Xiong Ma

**Affiliations:** ^1^Division of Gastroenterology and Hepatology, Key Laboratory of Gastroenterology and Hepatology, Ministry of Health, State Key Laboratory for Oncogenes and Related Genes, Renji Hospital, Shanghai JiaoTong University School of Medicine, Shanghai, China; ^2^Department of Gastroenterology and Hepatology, The Hubei Clinical Center and Key Laboratory of Intestinal and Colorectal Diseases, Zhongnan Hospital, Wuhan University, Wuhan, China; ^3^Department of Gastroenterology and Hepatology, Nantong Institute of Liver Disease, Third Affiliated Hospital of Nantong University, Nantong, China; ^4^Division of Rheumatology, Allergy, and Clinical Immunology, University of California, Davis, Davis, CA, United States

**Keywords:** mucosal-associated invariant T cell, nonalcoholic fatty liver disease, macrophage, cytokine, MR1

## Abstract

Mucosal-associated invariant T (MAIT) cells, a novel population of innate-like lymphocytes, have been involved in various inflammatory and autoimmune diseases. However, their role in the development of nonalcoholic fatty liver disease (NAFLD) remains unclear. In this study, we investigated the alterations of phenotype and immunological function of MAIT cells in NAFLD. Analysis of PBMCs in 60 patients with NAFLD and 48 healthy controls (HC) revealed that circulating MAIT cell frequency decreased in NAFLD, especially in the patients with higher serum levels of γ-glutamyl transferase or total triglyceride. Functional alterations of circulating MAIT cells were also detected in NAFLD patients, such as the increased production of IL-4 whereas the decreased production of IFN-γ and TNF-α. Furthermore, elevated expression of CXCR6 was observed in circulating MAIT cells of patients. Meanwhile, we found an increased number of MAIT cells in the livers of NAFLD, and the number was even greater in patients with higher NAFLD activity score. Moreover, activated MAIT cells induced monocytes/macrophages differentiation into M2 phenotype *in vitro*. Additionally, MAIT cells were enriched and displayed Th2 type cytokines profile in livers of wild type mice fed with methionine and choline deficient diet (MCD). Notably, mice deficient of MAIT cells exhibited more severe hepatic steatosis and inflammation upon MCD, accompanied with more CD11c^+^ proinflammatory macrophages (M1) and less CD206^+^ anti-inflammatory macrophages (M2) in livers. Our results indicate that MAIT cells protect against inflammation in NAFLD through producing regulatory cytokines and inducing anti-inflammatory macrophage polarization, which may provide novel therapeutic strategies for NAFLD.

## Introduction

Nonalcoholic fatty liver disease (NAFLD) is one of the most common chronic liver diseases worldwide, with the global prevalence of 25.24% (1). It covers a spectrum of diseases including simple steatosis, nonalcoholic steatohepatitis (NASH), liver cirrhosis, and hepatocellular carcinoma. Although the etiology of NAFLD is multi-factorial and remains largely enigmatic, it is well accepted that inflammation is a central component of NAFLD development and progression (2). The immune response contributes critically to NAFLD progression, as the infiltrated innate and adaptive immune cells may accelerate and magnify the extent of liver injury (3). Thus, a better understanding of the immune pathways underlying NAFLD pathogenesis is vitally important for developing novel therapeutic strategies.

Mucosal-associated invariant T (MAIT) cells are a recently identified population of innate-like lymphocytes. Like invariant natural killer (iNKT) cells, MAIT cells exhibit restricted T cell receptor (TCR) diversity and express the semi-invariant TCR Vα7.2-Jα33 in humans and Vα19-Jα33 in mice with a limited set of Vβ chains (4, 5). MAIT cells can recognize bacterial metabolites deriving from the biosynthesis of riboflavin, such as 5-(2-oxopropylideneamino)-6-D-ribityl-aminouracil (5-OP-RU) and 5-(2-oxoethylideneamino)-6-D-ribityl-aminouracil (5-OE-RU), which can be presented by MHCI-related molecule (MR1) in antigen presenting cells (APCs) (6). Furthermore, the soluble tetramerized MR1 molecules, refolded with 5-OP-RU Ag, are capable of detecting all MAIT cells (7). MAIT cells constitute 1–10% of all αβT cells in human peripheral blood. They can also be found in peripheral tissues such as the mucosa of the intestine and lung, and are particularly abundant in the liver (8, 9). However, MAIT cells are present in very small number in laboratory mice and their population is equivalent to 0.6% of T cells in the liver of B6 mouse (10). MAIT cells can produce IFN-γ, TNF-α, IL-4, IL-10, and IL-17, therefore they are a major source of Th1, Th2, and Th17 cytokines (11). Previous studies have shown alterations of MAIT cell frequency in various diseases: infectious diseases, autoimmune disease, obesity, and diabetes (12–15). Nevertheless, the role of MAIT cells in NAFLD is still unknown and yet to be unraveled. Here, we investigated the phenotype, clinical relevance and biological functions of MAIT cells in NAFLD patients. In addition, we explored the immunological role that MAIT cells played in NASH pathogenesis *in vitro* as well as in mouse model.

## Materials and methods

### Healthy controls and patients

Peripheral blood were collected from 60 patients with NAFLD between January 2016 and April 2017 in Renji Hospital, Shanghai Jiao Tong University School of Medicine. The diagnosis of NAFLD was based on the criteria established by Chinese National Work-shop on Fatty Liver and Alcoholic Liver Disease (16). Forty-eight healthy volunteers matched by age and gender were enrolled as controls. Paraffin-embedded liver tissues were also studied, which were derived from 40 NAFLD patients through ultrasound-guided needle liver biopsies. The histological sections were stained with hematoxylin and eosin (HE). And liver tissues were collected as controls from 5 healthy donors whose livers would be subsequently used for transplantation. The clinical characteristics of the subjects were described in Table 1. The study was approved by the Ethics Committee of Renji Hospital. All subjects gave written informed consent in accordance with the Declaration of Helsinki.

**Table 1:** Characteristics of subjects in this study.

	**Health controls**	**NAFLD patients**
Cases	48	60
Male/Female	36/12	46/14
Age (years)	45.42 ± 2.3	46.02 ± 3.1
Body Mass Index (kg/m^2^)	21.77 ± 0.44	27.71 ± 0.37^***^
γ-glutamyl transferase (IU/L)	16.57 ± 1.47	92.27 ± 9.06^***^
Alanine aminotransferase (IU/L)	14.70 ± 1.07	50.78 ± 4.15^***^
Triglyceride (mmol/L)	1.01 ± 0.56	3.67 ± 0.41^***^
Total cholesterol (mmol/L)	3.90 ± 0.20	5.30 ± 0.16^***^
HbA1c (%)	5.34 ± 0.06	6.22 ± 0.19^**^
Fasting glucose (mmol/L)	5.01 ± 0.07	6.14 ± 0.27^**^

*** P < 0.001,

** *P < 0.01*.

### Flow cytometric analysis

Peripheral blood mononuclear cells (PBMCs) were obtained by Ficoll-Paque (GE Healthcare, USA) gradient centrifugation. Cell surface and intracellular staining was performed using the following monoclonal antibodies (mAbs): anti-TCR-Vα7.2 (Biolegend, USA), anti-CD3, anti-CD161, anti-CD69, anti-PD-1, anti-TNFα, anti-IFN-γ, anti-IL-4, anti-IL-10, anti-CCR5, anti-CXCR6 (BD Biosciences, USA). For intracellular staining, the Cytofix/Cytoperm^TM^ Fixation/Permeabilization kit was utilized according to the manufacturer's specification (BD Biosciences, USA). Data were acquired by flow cytometry (BD LSRFortessa, USA), and analyzed with FlowJo software Version7.6 (Tree Star, Ashland, OR, USA).

### Immunohistochemistry and immunofluorescence

Paraffin-fixed tissue sections were soaked in citrate buffer and treated with microwave for antigen retrieval. After quenching endogenous peroxidase activity with 3% H_2_O_2_ in methanol, the sections were incubated in 5% bovine serum albumin (BSA) followed by incubation with antibodies against MR1 (1:300, monoclone 26.5, Santa Cruz) for immunohistochemistry, CD3 (1:200) and MR1 tetramer [TEM, 1:500, provided by the National Institutes of Health (NIH) tetramer facility] for immunofluorescence overnight at 4°C. After rinsing in phosphate-buffered saline three times, the sections were incubated with secondary antibody or fluorochrome-conjugated secondary antibody (1:300, Alexa Fluor 488 donkey anti-mouse) for 30 min at room temperature. 3′-diaminobenzidine (DAB) was used for visualization in immunohistochemistry. The nucleus was counterstained by hematoxylin or 4′,6-diamidino-2-phenylindole (DAPI). Lastly, histological immunofluorescence was observed by Carl Zeiss LSM 710 laser scanning (confocal) system and LSM Image Browser software (Carl Zeiss, Inc., Germany).

### Measurement of MR1 expression

Monocytes were sorted out from human PBMCs using anti-CD14 microbeads and maintained at 2 × 10^5^ cells/ml in RPMI 1640 medium supplemented with 10% fetal bovine serum (FBS), 100 mg/ml streptomycin, and 100 U/ml penicillin. Under stimulation with macrophage colony-stimulating factor (M-CSF, 100 ng/ml) for 3 days, monocytes differentiated into macrophages (17). Then free fatty acid (FFA, mixture of oleic acid and palmitic acid at a ratio of 2:1, dissolved in BSA) or BSA as control was added to stimulate CD14^+^ macrophages for 6 h. To stain for MR1, PE-anti-MR1 was added at 10 μg/ml for another 4-h culture (18). After digested by Trypsin-EDTA, these macrophages were analyzed by flow cytometry.

### Cell purification and co-culture

Monocytes were sorted out from PBMCs in healthy control using anti-CD14 microbeads and MAIT cells were sorted out using Vα7.2^+^ microbeads by magnetic cell separation. Vα7.2^+^ cells were activated with anti-CD3/CD28-coupled beads (1:2 bead/cell ratio, Miltenyi Biotec). After cultured in medium with M-CSF (100 ng/ml) for 24 h, monocytes (2 × 10^5^ cells/ml) were co-cultured with activated MAIT cells (at a monocyte/MAIT cell ratio of 2:1) or unactivated MAIT cells for 3 days. Transwell experiments were performed in 24-well plates (0.4 μm pore size) with monocytes/macrophages cultured in the lower wells and MAIT cells in the inserts. Adherent macrophages were digested and collected for labeling of anti-HLA-DR, anti-CD80, anti-CD86, anti-CD163, and anti-CD206 antibodies (Biolegend, USA).

### Cytometric bead array (CBA)

PBMCs (1 × 10^6^ cells/ml) and sorted Vα7.2^+^ cells (2 × 10^5^ cells/ml) were cultured in RPMI-1640 supplemented with 10% FBS. After PBMCs were stimulated with PMA-ionomycin for 6 h and sorted Vα7.2^+^ cells were activated with anti-CD3/CD28-coupled beads for 96 h, culture supernatant were collected for IL-4 quantification. The level of IL-4 was determined by CBA according to the manufacturer's protocol (BD Biosciences). The concentrations were quantified using FACP Array software v3.0.

### Mice

Wild type (WT) and MR1-knockout (MR1^−/−^, lack of MAIT cells, obtained from Shanghai Biomodel Organisms Center, Inc) male mice (C57BL/6 background) were used to establish NASH model. Mice were fed *ad libitum* for 4 weeks either with normal diet (ND) or with methionine and choline deficient diet (MCD, Research Diets, USA) since the age of 8 weeks. Mice were housed in a specific pathogen-free (SPF) facility and fresh food was provided on a weekly basis. Blood was collected for alanine aminotransferase (ALT) measurement and liver tissue were collected for histology, biochemical determination as well as RNA isolation. This study was carried out in accordance with the recommendations of *Guide for the Care and Use of Laboratory Animals*, Ethics Committee of Renji Hospital. The protocol was approved by the Ethics Committee of Renji Hospital.

### Hepatic mononuclear cells (HMNCS) isolation and labeling

HMNCs were isolated as described previously (19). Briefly, livers were perfused with sterile saline solution to remove blood cells and then were homogenized in a Stomacher80 homogenizer for 2 min. After the liver homogenate passing through a 100-μm filter, hepatic non-parenchymal cells were collected by centrifugation at 450 g for 5 min. Subsequently, the mononuclear cell fraction was isolated using a gradient of 35% Percoll (GE Healthcare, USA) at 900 g for 30 min. After incubated with mouse BD Fc BlockTM for 30 min, the HMNCs were labeled with cell surface markers including MR1-5-OP-RU TEM (provided by the NIH tetramer facility) as well as anti-mouse CD3, TCRβ, CD45, F4/80, CD11c, and CD206 mAbs, and intracellular markers including anti-mouse IFN-γ, IL-4 together with IL-10 mAbs. Fixable Viability Stain was used to discern dead cells.

### Statistical analysis

Statistical evaluations were performed with GraphPad Prism 5.0 (GraphPad Software Inc, USA) and SPSS19.0 (SPSS Inc, USA). Data was expressed as mean ± standard error (SE). Mann–Whitney *U*-test and paired *t*-test were used to analyze differences in continuous variables. Correlations were determined by Spearman's correlation coefficient. One-way analysis of variance (ANOVA) followed by *post hoc* Bonferroni test was used for multiple comparisons. In all tests, *P* < 0.05 was considered as statistically significant. Animal experiments were repeated at least two times on two separate occasions.

## Results

### MAIT cell frequency among circulating CD3^+^ T cells was lower and correlated with clinical parameters in patients with NAFLD

**Figure 1:**
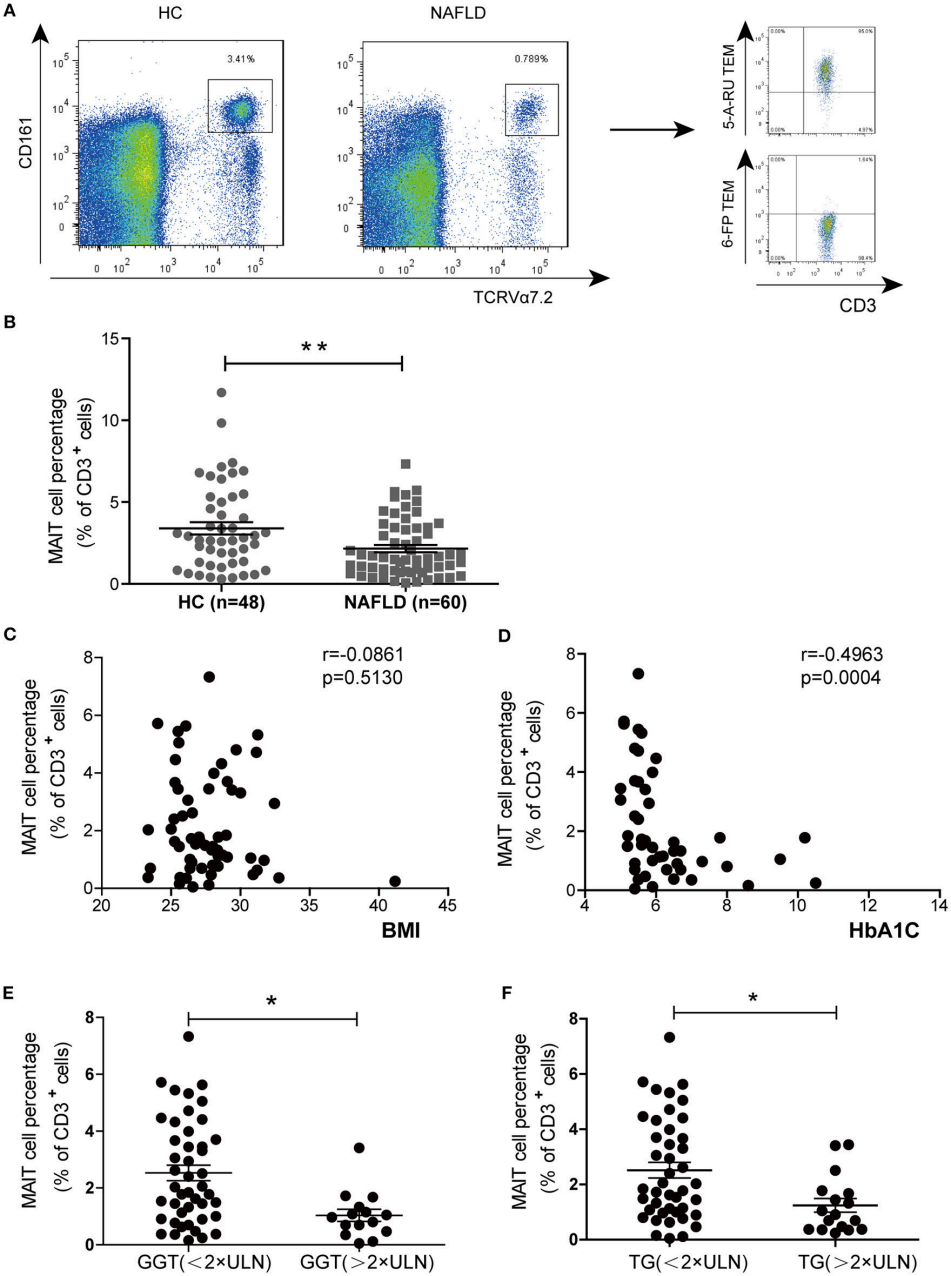
MAIT cell percentages among circulating CD3^+^ T cells in HC and NAFLD patients, as well as correlations between circulating MAIT cell percentage and clinical parameters in NAFLD patients. **(A)** Representative flow cytometry scatter plots from HC and NAFLD patient (Left panel). CD3^+^CD161^high^Vα7.2^+^ cells were confirmed by MR1-5-OP-RU TEM and MR1-6-FP TEM (negative control) (Right panel). **(B)** Statistical analysis of circulating MAIT cell frequency in HC (*n* = 48) and patients with NAFLD (*n* = 60). Spearman correlation between MAIT frequency with **(C)** HbA1c (*n* = 47) and **(D)** BMI (*n* = 60) in NAFLD patients. **(E,F)** NAFLD patients with higher serum GGT or TG (> 2 × ULN) had lower peripheral MAIT cell percentage than those patients with lower (< 2 × ULN) GGT or TG. Data were analyzed with Mann–Whitney *U*-tests. *P < 0.05, **P < 0.01. TEM, tetramer; BMI, body mass index; GGT, γ-glutamyl transferase; TG, triglyceride; ULN, upper limit of normal.

We examined MAIT cell percentages among peripheral blood CD3^+^ T cells in 60 NAFLD patients and 48 HC by FACS analysis. The frequency of circulating MAIT cells (defined as CD3^+^CD161^high^TCR Vα7.2^+^) was significantly lower in NAFLD patients compared to HC (Figures 1A,B). We then confirmed the finding by using human MR1 tetramers (TEM), which can detect MAIT cells specifically. Most (>95%) CD3^+^CD161^high^TCR Vα7.2^+^ cells were bound by MR1-5-OP-RU TEM (non-antigenic MR1-6-formylpterin (6-FP) TEM used as negative control) (Figure 1A). Furthermore, we investigated whether circulating MAIT cells frequency was associated with clinical parameters in NAFLD patients. The results showed a negative correlation between MAIT cell frequency and HbA1c level, but not with body mass index (BMI) (Figures 1C,D). In addition, circulating MAIT cell frequency was lower in NAFLD patients with higher serum γ-glutamyl transferase (GGT) or triglyceride (TG), than those with lower GGT or TG (Figures 1E,F). This indicates that the frequency of circulating MAIT cell is inversely correlated with the severity of NAFLD.

### More circulating MAIT cells were activated and the immune functions of MAIT cells altered in NAFLD patients

**Figure 2:**
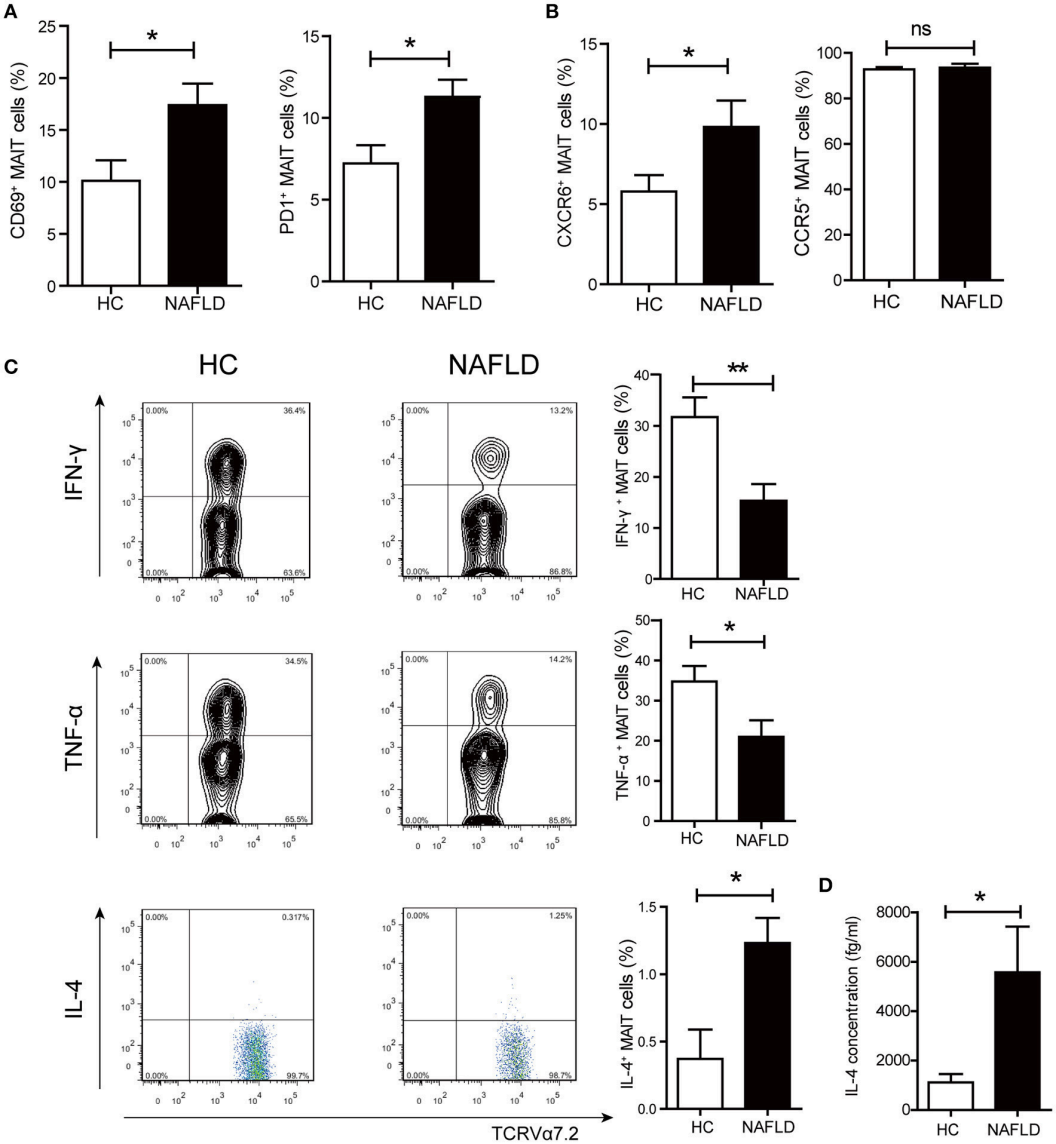
Phenotype and function alterations of circulating MAIT cells in NAFLD patients. **(A)** CD69^+^ and PD-1^+^ expression on MAIT cells in HC and NAFLD patients. **(B)** Higher frequency of CXCR6^+^ MAIT cells in NAFLD patients compared to HC, and high frequency of CCR5^+^ MAIT cells in both 2 groups without statistical difference. **(C)** Representative examples of intracellular cytokines staining (Left panel) and the frequency of MAIT cells from HC and NAFLD patients that produced IFN-γ, TNF-α or IL-4 after stimulation with PMA-ionomycin (Right panel). (HC, *n* = 20; NAFLD patients, *n* = 25). **(D)** IL-4 concentrations in PBMCs supernatant from HC and NAFLD patients with PMA-ionomycin stimulation. (*n* = 15). Data were analyzed with Mann–Whitney *U*-test.*P < 0.05, **P < 0.01, ns: no statistical significance.

Next, we investigated the activation and cytokine production of circulating MAIT cells from HC and patients with NAFLD. The frequency of MAIT cells expressing CD69 (early activation marker) and PD-1 (late activation marker) were higher in NAFLD patients compared to HC (Figure 2A). Previous reports have demonstrated that chemokine receptor CXCR6 was involved in lymphocyte recruitment to liver (20). Here we observed that MAIT cells expressed higher level of CXCR6 in NAFLD patients compared with HC, suggesting that MAIT cells of NAFLD patients had a stronger tendency to migrate to the liver (Figure 2B). Additionally, circulating MAIT cells in both NAFLD patients and HC showed high frequency of CCR5 expression, indicating their strong ability to migrate to tissues such as liver and adipose tissue (21). No intracellular IFN-γ, TNF-α, or IL-4 were detected in the absence of *ex vivo* stimulation, indicating that these cytokines were not produced by MAIT cells *in vivo*. However, analysis of cytokine secretion after PMA-ionomycin stimulation revealed that the frequency of circulating MAIT cells producing IFN-γ and TNF-α were significantly lower in NAFLD patients, whereas the frequency of IL-4-producing MAIT cells was higher (Figure 2C). However, there was no significant difference in the frequency of IL-10^+^ MAIT cells between the two groups (Supplementary Figure 1A). Meanwhile, we evaluated IL-4 production in supernatant of PBMCs by CBA analysis after stimulation. Compared with PBMCs from HC, NAFLD patients' PBMCs showed a higher concentration of IL-4 (Figure 2D).

### Increased MAIT cells in livers of NAFLD patients and MR1 expression in kupffer cells

**Figure 3:**
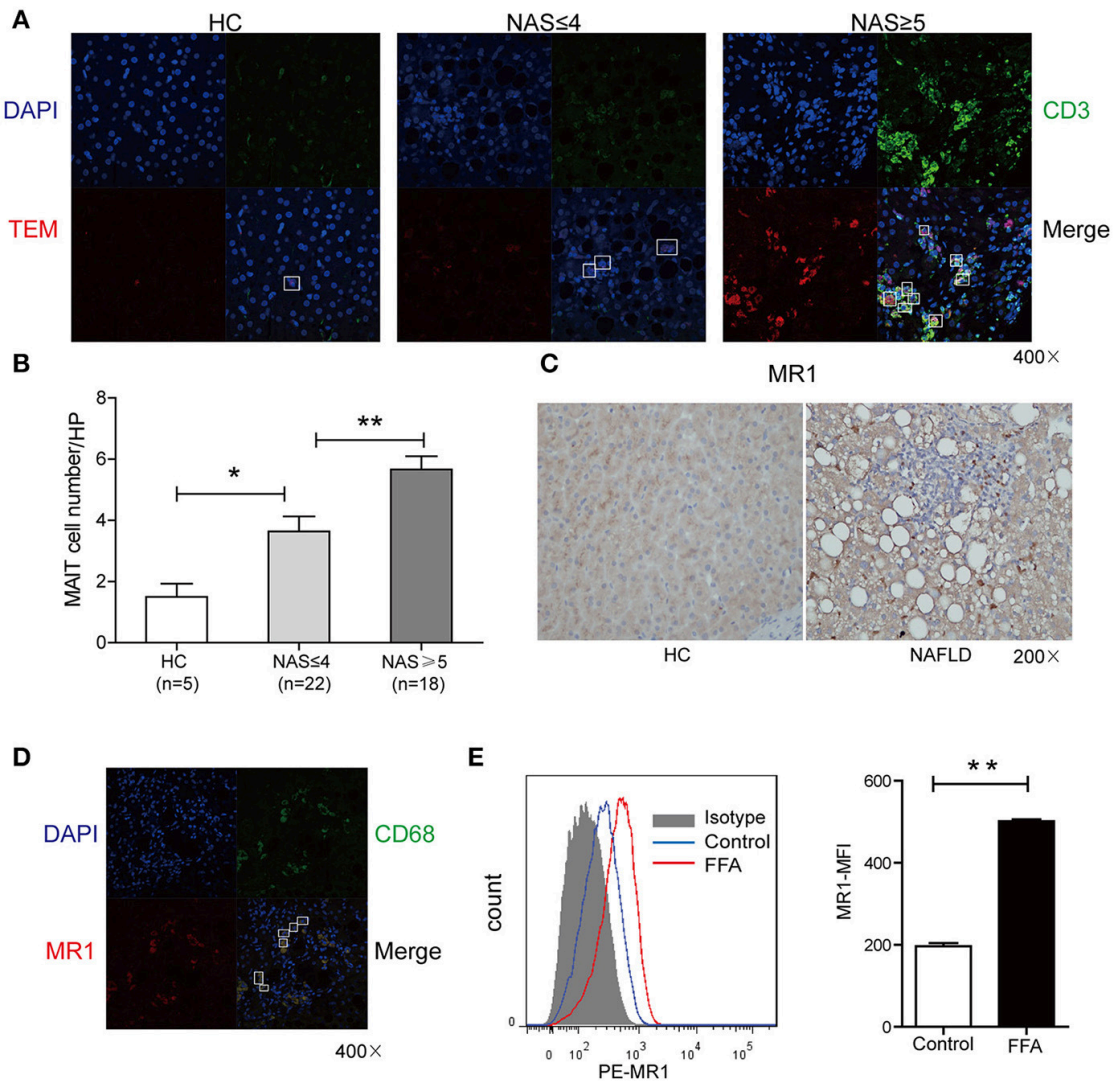
Comparisons of MAIT cell distribution and MR1 expression in livers between patients and HC. **(A)** Representative confocal staining of CD3 (in green), MR1-5-OP-RU TEM (in red) and DAPI (for nuclei in blue) in the livers of HC, and patients with NAS ≤ 4 (*n* = 22), NAS ≥ 5 (*n* = 18) (original magnification, × 400). **(B)** The number of hepatic MAIT cells in NAFLD patients increased compared with HC and was positively correlated with NAS in NAFLD patients. **(C)** Representative immunohistochemical staining of MR1 in liver of HC and NAFLD patients (original magnification, × 200). **(D)** Representative confocal staining of CD68^+^ (in green), MR1 (in red) and DAPI (for nuclei in blue) (original magnification, × 400). **(E)** Representative overlaid histogram plots of MR1 staining of CD14^+^ monocytes-derived macrophages with FFA stimulation and BSA as control (Left panel). Statistical analysis of MFI of MR1 staining depicted an elevated level of MR1 expression on macrophages after FFA stimulation (Right panel) (*n* = 5). Data were analyzed with Mann–Whitney *U-*test. *P < 0.05, **P < 0.01. NAS, NAFLD activity score; FFA, free fatty acid; BSA, bovine serum albumin; MFI, mean fluorescence intensity.

*In situ* staining was used to determine the localization and distribution of MAIT cells in liver. The MAIT cells were defined by immunofluorescent double-staining for CD3 and MR1-5-OP-RU tetramer. In healthy liver, MAIT cells scattered closely to and within portal area, while MAIT cells accumulated around degenerated hepatocytes with fat deposits in the sinusoidal areas in the livers of NAFLD patients (Figure 3A). Moreover, the number of MAIT cells positively correlated with NAFLD activity score (NAS) in NAFLD patients (Figure 3B). We next investigated the expression of MR1 in livers of NAFLD patients and HC by immunohistochemistry. MR1 expressed in cytoplasm of hepatocytes in liver of healthy control, while in liver of patients, MR1 was observed in inflammatory cells (Figure 3C). We further identified that MR1 mainly expressed in CD68^+^ Kupffer cells by immunofluorescence (Figure 3D). It has been reported that when APCs are stimulated, MR1 molecule traffics to the cell surface from the endoplasmic reticulum to present antigen to MAIT cells (22). To study if FFA can induce MR1 expression on the surface of Kupffer cells, we stimulated CD14^+^ monocytes-derived macrophages with long chain fatty acids *in vitro* (23). We found FFA at the concentration of 0.5 mmol/L increased MR1 expression on the surface of macrophages (Figure 3E), suggesting that Kupffer cells are potential to activate MAIT cells in an MR1-dependent manner.

### Activated MAIT cells could induce M2 macrophages polarization *in vitro*

**Figure 4:**
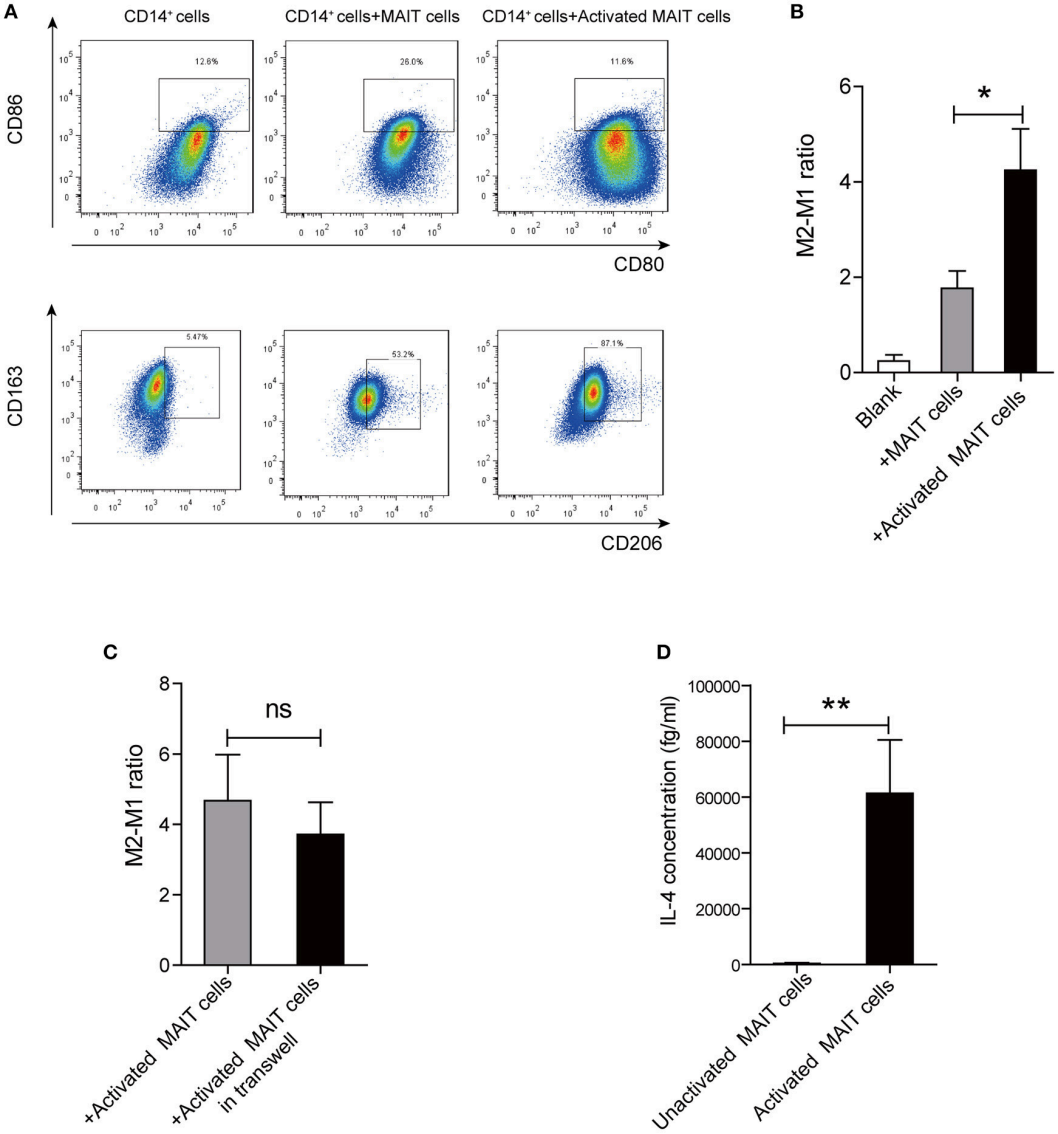
Macrophage polarization induced by activated MAIT cells *in vitro*. **(A)** Representative flow cytometry scatter plots of M1 (HLADR^+^CD80^+^CD86^+^) and M2 (CD163^+^CD206^+^) phenotype in CD14^+^ monocytes treated with unactivated MAIT cells or activated MAIT cells. **(B)** A higher M2/M1 ratio in macrophages co-cultured with activated MAIT cells (*n* = 5). **(C)** No significant difference in M2/M1 cells ratio among macrophages treated with activated MAIT cells between co-culture system and transwell experiments (*n* = 4). **(D)** Comparison of IL-4 production in unactivated and activated MAIT cells (*n* = 4). Data were analyzed with paired *t*-test and Mann–Whitney *U*-test. *P < 0.05, **P < 0.01, ns: no statistical significance.

To investigate whether MAIT cells can induce the differentiation of monocytes into anti-inflammatory macrophages, we cultured CD14^+^ monocytes alone, or with Vα7.2^+^ cells unactivated/activated by anti-CD3/CD28-coupled beads. Interestingly, compared with monocytes treated with unactivated-MAIT cells, monocytes treated with activated-MAIT cells displayed a high percentage of CD163^+^CD206^+^ cells (M2 type) and a low percentage of HLADR^+^CD80^+^CD86^+^ cells (M1 type) (Figure 4A). And M2/M1 cells ratio was higher among macrophages co-cultured with activated-MAIT cells (Figure 4B). To investigate whether the effect of MAIT cells on M2 polarization was caused by cell contact or soluble molecules, we performed additional transwell experiments. The results showed that M2/M1 cells ratio among macrophages treated with activated MAIT cells did not decrease in transwell system (Figure 4C), which indicated that regulation of macrophage polarization by MAIT cells mostly depended on soluble factors. Additionally, we analyzed the IL-4 production of sorted Vα7.2^+^ cells and found there was a significant release of IL-4 after activation (Figure 4D).

### MAIT cells were enriched and displayed Th2 type cytokines profile in livers of MCD mice

**Figure 5:**
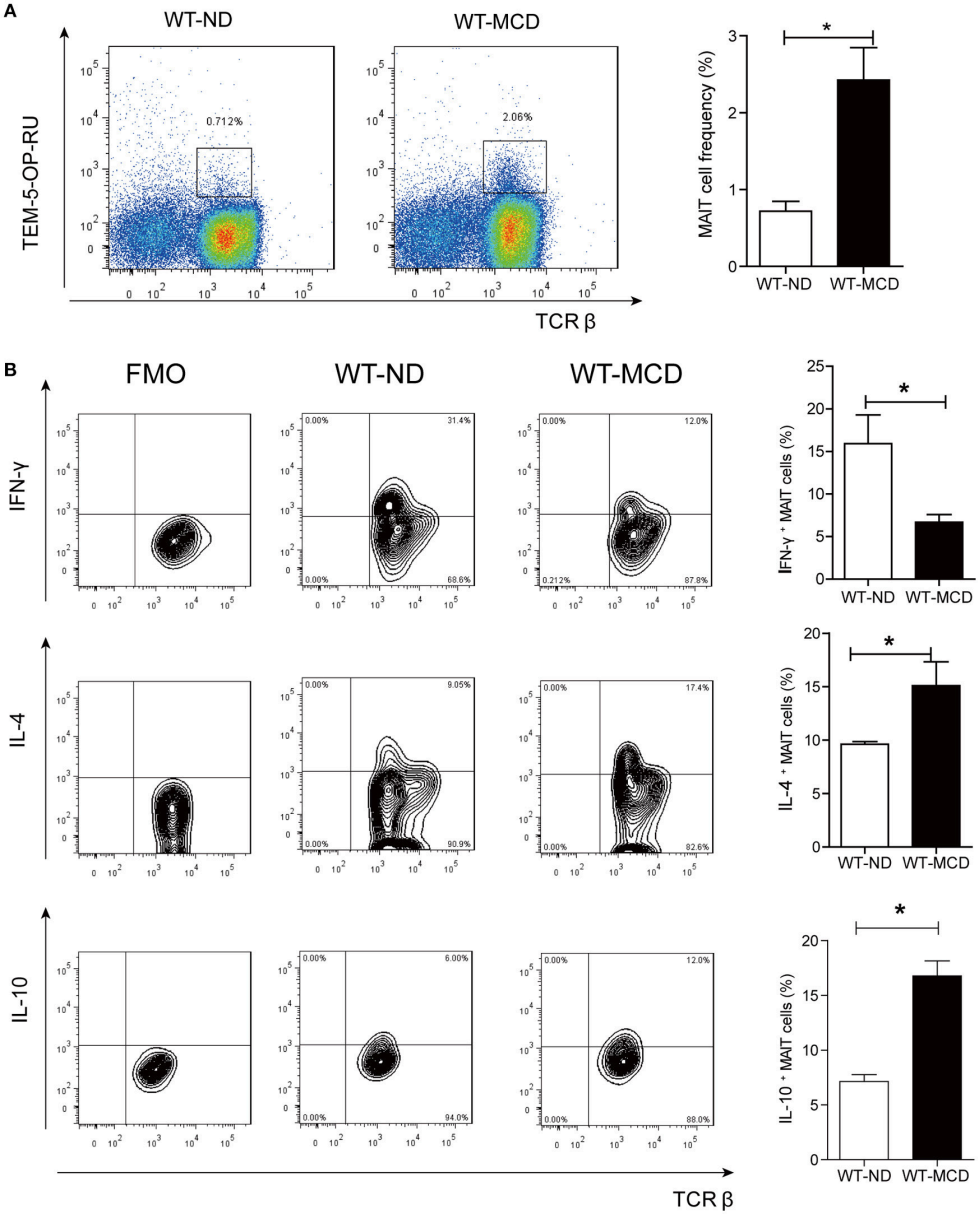
Frequency and cytokines profile of MAIT cell in livers of WT-ND mice and WT-MCD mice. **(A)** The frequency of MAIT cells in liver from WT mice on ND and MCD. **(B)** The percentage of PMA-ionomycin-stimulated intra-hepatic MAIT cells that produce IFN-γ, IL-4, or IL-10 in WT mice upon ND and MCD (*n* = 6). Data were analyzed with Mann–Whitney *U*-test. *P < 0.05. WT, wild type; ND, normal diet; MCD, methionine and choline deficient diet; TEM, tetramer.

As a diet that impairs hepatic beta-oxidation, MCD is widely used to establish animal model of NASH, for it causes more severe inflammation, oxidative stress and apoptosis in the liver (24). Thus, we evaluated alterations of hepatic MAIT cells in mouse model by MCD feeding. In mice, MAIT cells were defined as CD3^+^TCRβ^+^MR1-5-OP-RU-TEM^+^ cells (Figure 5A) and MR1-6-FP-TEM was used as negative control (Supplementary Figure 2A). After 4-weeks-MCD feeding, the frequency of intra-hepatic MAIT cells was higher in MCD mice than that in ND mice (Figure 5A). Of note, there were more IL-4^+^ and IL-10^+^ cells as well as less IFN-γ^+^ ones among hepatic MAIT cells in WT-MCD mice compared with WT-ND mice (Figure 5B).

### MAIT cell deficiency triggered progression of steatohepatitis with more proinflammatory cytokine and macrophages

**Figure 6:**
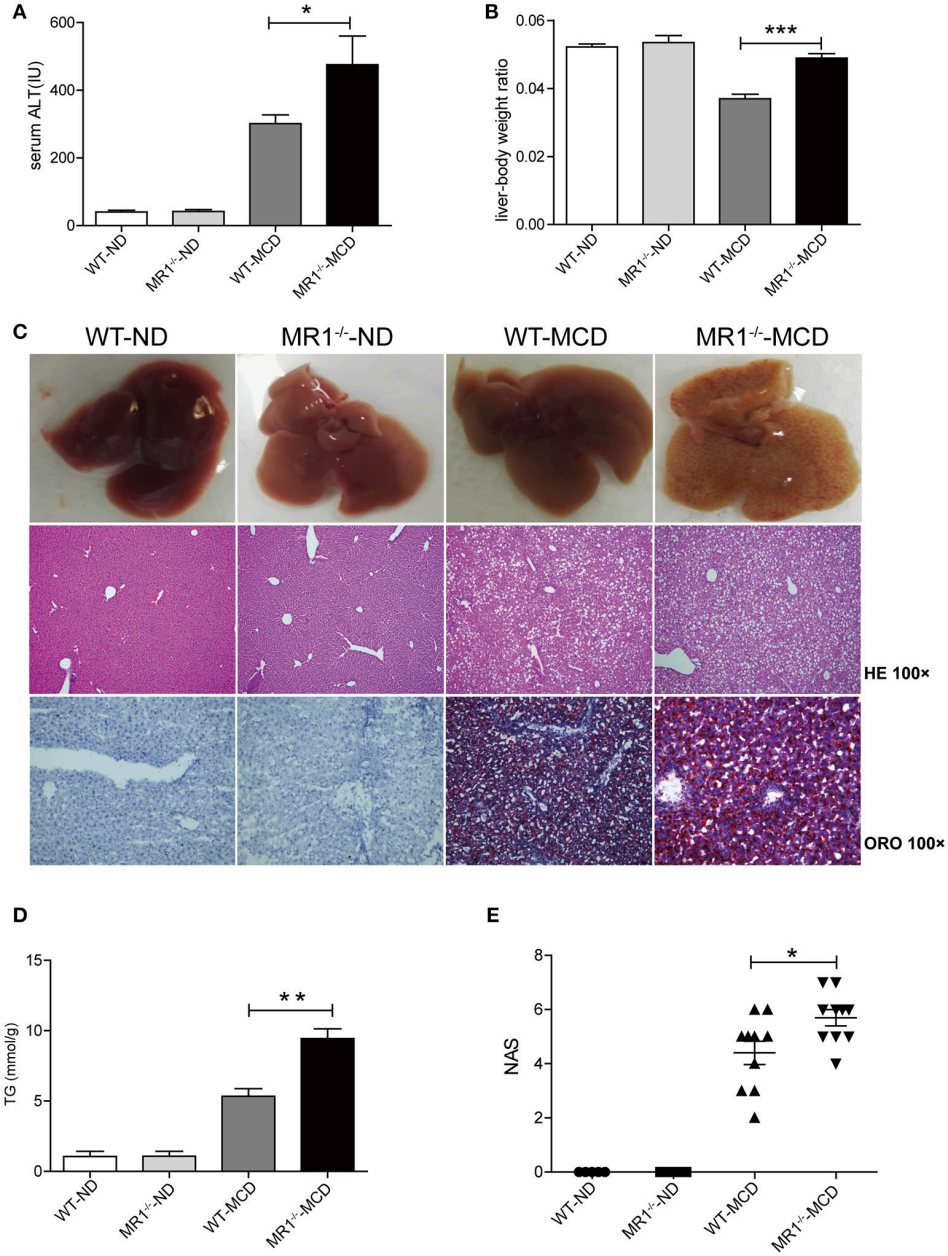
More severe hepatic steatosis and inflammation in MR1^−/−^ -MCD mice compared with WT-MCD mice. WT serum ALT levels **(A)** and liver-to-body weight ratio **(B)** in WT and MR1^−/−^ mice on ND and MCD. **(C)** The gross picture, HE and ORO staining of livers from WT and MR1^−/−^ mice on ND and MCD (original magnification, × 100) (*n* = 10). TG level **(D)** and NAS **(E)** of livers in mice in WT and MR1^−/−^ mice upon ND and MCD (*n* = 10). Data were analyzed using ANOVA with Bonferroni test comparison. *P < 0.05, **P < 0.01, ***P < 0.001. ALT, alanine aminotransferase; HE, hematoxylin and eosin; ORO, oil red O; TG, triglyceride; NAS, NAFLD activity score.

**Figure 7:**
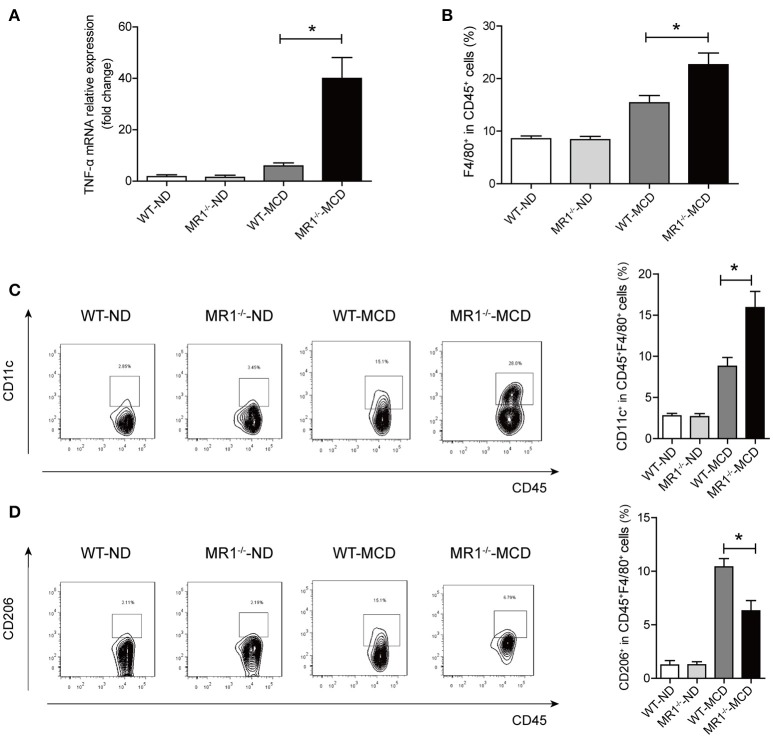
Comparisons of pro-inflammatory cytokine expression and macrophages in livers between MR1^−/−^-MCD mice and WT-MCD mice. **(A)** Hepatic expression of the TNF-α mRNA in WT and MR1^−/−^ mice on ND and MCD. **(B)** Higher total macrophage (F4/80^+^) number in liver of MR1^−/−^ mice on MCD compared to WT-MCD mice. Representative dot plots and the percentage of CD11c^+^ macrophages **(C)** and CD206^+^ macrophages **(D)** from individual mice in WT and MR1^−/−^ group on ND or MCD (*n* = 10). Data were analyzed using ANOVA with Bonferroni test comparison. *P < 0.05.

We examined NASH phenotype in MR1^−/−^ mice (lack of MAIT cells, Supplementary Figure 2B). Upon MCD feeding, MR1^−/−^ mice displayed a higher serum ALT level and a higher liver-to-body weight ratio than that of WT mice (Figures 6A,B). Furthermore, MCD feeding induced more severe hepatic steatosis in MR1^−/−^ mice compared to WT mice, evident by increased lipid accumulation shown by HE and oil red O (ORO) staining as well as hepatic TG levels (Figures 6C,D). In addition, MR1^−/−^ mice had a higher histopathological NAS (Figure 6E) and a higher gene expression of proinflammatory cytokine TNF-α, which is the classical cytokine produced by M1 macrophages (Figure 7A) (25). Likewise, there were more macrophages (F4/80^+^) in the livers of MR1^−/−^-MCD mice, compared to WT-MCD mice (Figure 7B). Importantly, in MR1^−/−^-MCD mice, hepatic macrophages polarized into more CD11c^+^ M1 (pro-inflammatory phenotype) and less CD206^+^ M2 (anti-inflammatory phenotype) (Figures 7C,D).

## Discussion

**Figure 8:**
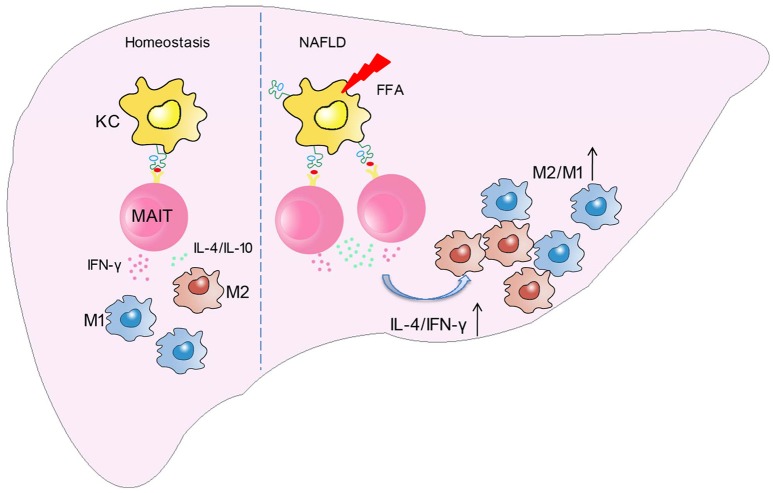
Schematic diagram illustrating the role of MAIT cells in homeostasis and NAFLD. In the development of NAFLD, FFA induces MR1 expression increase in kupffer cells (KC). Circulating MAIT cells are recruited to the liver and then activated in MR1-dependent manner, secreting more Th2 type cytokines with less Th1 type cytokine. Consequently, activated MAIT cells induce M2 macrophage polarization, providing protection against inflammation in NASH.

In this study, we demonstrated for the first time that the frequency and function of MAIT cells altered in patients with NAFLD, and MAIT cells played a protective role in the pathogenesis of NASH by regulating macrophage polarization. More specifically, FFA induces MR1 expression increase in Kupffer cells (KC). Circulating MAIT cells are recruited to the liver and then activated in MR1-dependent manner, secreting more Th2 type cytokines with less Th1 type cytokine. Consequently, activated MAIT cells induce M2 macrophage polarization, providing protection against inflammation in NASH (Figure 8).

Decreased frequency of circulating MAIT cells has been described in various non-infectious diseases: systemic lupus erythematosus (SLE) (26), inflammatory bowel disease (IBD) (27), autoimmune liver diseases (28, 29), diabetes and obesity (14). In these diseases, reduced circulating MAIT cells are accompanied by increased MAIT cells in synovial fluid, inflamed gut mucosa, liver portal tracts, and adipose tissue. Meanwhile, MAIT cells express more chemokine receptors such as CCR6 (30), CXCR6, integrin αEβ7, CXCR3 (31). The results above suggest that, in these diseases, circulating MAIT cells may be recruited to inflamed tissues. Similar results were obtained in our study that MAIT cell frequency decreased in peripheral blood of NAFLD patients, while the frequency of MAIT cells in the liver increased, which was positively correlated with NAS. WT mice upon MCD also showed higher MAIT cell frequency in livers. Exceptionally, several studies reported that intestinal and hepatic MAIT cells were depleted respectively in IBD and chronic liver inflammation. They believed that MAIT cells were recruited from the blood to the gut or liver where they were activated by inflammatory cytokines and further underwent activation-induced cell death (28, 32, 33). The discrepancy in results of different studies may be attributed to some factors, such as different disease stages when samples were collected, different locations where samples were collected and different analytical methods (e.g., direct cell count by immunohistochemistry and immunofluorescence, or proportion evaluation through flow cytometry analysis).

MAIT cells were activated and functionally exhausted in patients, defined by increased expression of CD69 and PD-1, which was in line with the previous studies (26, 30, 34, 35). It was reported that MAIT cells less frequently produced IFN-γ but more frequently produced IL-17 in many diseases, exhibiting a shift from Th1 type to Th17 type cytokine profile (27, 34, 36). Besides, MR1-restricted Vα19i T cells can secrete IFN-γ, IL-4, IL-5, and IL-10 following TCR-mediated activation in transgenic mice (37). Our study demonstrated that circulating MAIT cells produced more IL-4, IL-17 (Supplementary Figure 1B) as well as less IFN-γ and TNF-α in NAFLD patients. Furthermore, hepatic MAIT cells in MCD mice displayed an elevated frequency of IL-4^+^ and IL-10^+^ cells together with a decreased frequency of IFN-γ^+^ cells, indicating a shift from Th1 type to Th2 type cytokine profile. The result may suggest that MAIT cells possess distinct biological functions in peripheral blood and local tissues.

In infectious diseases, APCs can present microbial-derived vitamin B metabolites via MR1 to TCR of MAIT cells. APCs also produce cytokines such as IL-12 and IL-18 that can activate MAIT cells in an antigen-independent mechanism (38). Early activation of MAIT cells is MR1-dependent, while their later activation involves both MR1-dependent and cytokine-dependent mechanism. We found that MR1 mainly expressed in CD68^+^ Kupffer cells in NAFLD patients and FFA stimulation can induce increased MR1 expression on the surface of macrophages *in vitro*, suggesting that FFA may regulate activation of MAIT cells. It has been found that FFA can bind to TLR4 on Kupffer cells to active inflammation pathways (39) and TLR signaling may be involved in MR1 expression on cell surface. Likewise, MR1 accumulation on the APC surface was reported to be dependent upon NF-κB signaling (18). Here, we speculate that APCs such as Kupffer cells might contribute to MAIT cells activation by increasing expression of MR1 on their surfaces. However, the mechanism underlying FFA-regulated MR1 expression needs further investigation.

Our MAIT cell-deficient MCD mice exhibited more severe hepatic steatosis and inflammation compared to WT mice, which means MAIT cells hold as protection against NASH. Multiple studies have suggested the involvement of hepatic macrophages in the progression of NASH (40). Classical CD11c-expressing M1 macrophages often aggregate around inflammation area and produce excess pro-inflammatory cytokines, such as IL-1β, IL-6, IL-8, IL-12 as well as TNF-α, and therefore play a pivotal role in inflammatory injury of tissue. By contrast, alternatively activated M2 macrophages (AAM) can generate anti-inflammatory cytokines, such as IL-10 and thus are involved in tissue repair and anti-inflammatory functions (41). It is well-known that macrophage polarization can be modified by T cells. IFN-γ produced by T cells enhances pro-inflammatory M1 macrophage differentiation, whereas IL-4, IL-13, and IL-10 promote anti-inflammatory M2 macrophage development (42, 43). A liver M2 macrophage polarization state is associated with reduced steatosis in humans and mice with NAFLD, and M2-conditioned medium promotes M1 macrophage apoptosis *in vitro* (41). We found that M1 polarization increased and M2 polarization decreased in MR1^−/−^ mice liver compared with WT counterparts on MCD, implying that MAIT cells may involve in the macrophage differentiation. Additionally, it was found *in vitro* that activated human MAIT cells produced an obvious increase of IL-4 and induced macrophage polarization to M2 phenotype. Since both IL-4 and IL-10 can induce M2 macrophage polarization, MAIT cells in liver may be more capable of promoting macrophage differentiation to M2 phenotype. Similarly with MAIT cells, iNKT cells, another conventional αβ T cells, has been reported to play the protective role in metabolic disorders. Activation of NKT cells with α-galactosylceramide can cause macrophages to polarize into anti-inflammatory M2 phenotype and improve glucose sensitivity (44).

Although High-Fat Diet (HFD) brings about a phenotype similar with the human disease, characterized by obesity, insulin resistance, NAFLD, and hyperlipidemia (45), we used MCD model for several reasons. The steatosis and inflammation in HFD model are substantially less remarkable than MCD model (46). In addition, alterations in gut microbiota also play an important role in HFD model. Thus, in order to focus on liver inflammation and avoid the confounding factor caused by intestinal microbiota, we choose MCD model in our study. However, the limitation of our study lies in the absence of rescue experiment performed through adoptive transfer, due to lack of Vα19i-overexpressing transgenic mouse. Besides, several studies have focused on the role of MAIT cells in liver fibrosis and cirrhosis. Bottcher et al. found the pro-fibrogenic role of MAIT cells in chronic liver disease and hepro-fibrogenic activation of human hepatic stellate cells by MAIT cells depended on IL-17A as well as direct cell-cell contact (28). Hegde et al. also identified activated MAIT cells as a novel major actor of the fibrogenic process. Using two models of chronic liver injury, they demonstrated that MAIT cell-enriched mice showed increased liver fibrosis and accumulation of hepatic fibrogenic cells, whereas MAIT cell-deficient mice are resistant (47). The role of MAIT cells in NASH-related fibrosis is not explored in this study and we believe it is worthy to be researched further.

In conclusion, our study provides a novel insight into the role of MAIT cells during NAFLD progression. It was revealed for the first time that there are alterations in MAIT cell frequency and function in NAFLD patients, and activated MAIT cells can promote anti-inflammatory M2 macrophage polarization. Given their potential to regulate immune response, MAIT cells may be a target for intervention in NAFLD.

## Author contributions

XM, MG, and RT designed and supervised the project. XM, JF, QW, and RT obtained funding. BH, JZ, and YW collected samples. YL, XJ, and ML contributed to data collection. YL, BH, JZ, and ZB contributed to perform the experiments. YL, WC, and YC performed bioinformatics and statistical analysis. YP and QM provide pathological support. RT and YL interpreted data. YL and BH drafted the manuscript. XM, MG, and RT revised the manuscript for important content.

### Conflict of interest statement

The authors declare that the research was conducted in the absence of any commercial or financial relationships that could be construed as a potential conflict of interest.

## Abbreviations

NAFLD: nonalcoholic fatty liver disease
NASH: nonalcoholic steatohepatitis
MAIT cell: mucosal-associated invariant T cell
iNKT cell: invariant natural killer cell
TCR: T cell receptor
MR1: MHCI-related molecule
APCs: antigen presenting cells
HE: hematoxylin and eosin
HC, health controls, PBMCs: peripheral blood mononuclear cells
BMI: body mass index
MCD: methionine and choline deficient diet
NAS: NAFLD activity score
ND: normal diet
ALT: alanine aminotransferase
WT: wild type
TEM: tetramer
GGT: γ-glutamyl transferase
TG: triglyceride
ULN: upper limit of normal
FFA: free fatty acid
IL: interleukin
IFN-γ, interferon-γ, TNF-α: tumor necrosis factor-α
HMNCs: hepatic mononuclear cells
AAM: alternatively activated macrophages
ORO: oil red O
M-CSF: macrophage colony-stimulating factor
HFD: high-fat Diet
DAPI, 4': 6-diamidino-2-phenylindole
FBS: fetal bovine serum
CBA: cytometric bead array
FMO: fluorescence minus one
MFI: mean fluorescence intensity.
