# Supplementary material for: Mucosal-Associated Invariant T Cells Improve Nonalcoholic Fatty Liver Disease Through Regulating Macrophage Polarization
Source: Front Immunol. 2018 Sep 4;9:1994. doi: 10.3389/fimmu.2018.01994 (PMC6131560; doi:10.3389/fimmu.2018.01994)
Supplement: Supplementary file 1 [file Data_Sheet_1.pdf]

Supplementary Material

Title

Mucosal-associated Invariant T Cells Improve Nonalcoholic Fatty Liver Disease through Regulating Macrophages Polarization

Authors

Yanmei Li, Bingyuan Huang, Xiang Jiang, Weihua Chen, Jun Zhang, Yiran Wei, Yong Chen, Min Lian, Zhaolian Bian, Qi Miao, Yanshen Peng, Jingyuan Fang, Qixia Wang, Ruqi Tang, M. Eric Gershwin, Xiong Ma

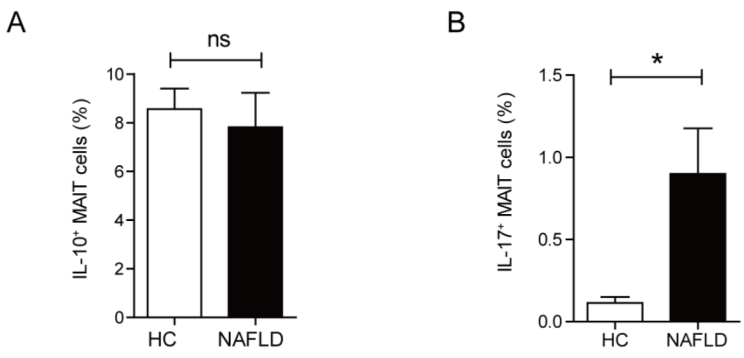

Supplementary Figure 1. The frequency of IL-10^{+} **(A)** and IL-17^{+} **(B)** MAIT cells in PBMCs from HC and NAFLD patients after stimulation with PMA-ionomycin (HC, n=20; NAFLD, n=25). Data were analyzed with Mann–Whitney U test. * *P*< 0.05; ns: no statistical significance.

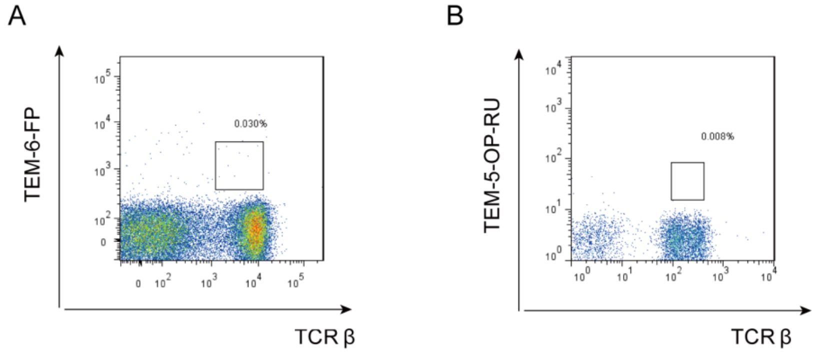

Supplementary Figure 2. (A) Representative scatter plot of MR1-6-FP-TEM^{+} as negative control in liver of WT mice. **(B)** No MAIT cells in liver of MR1$^{-/-}$ mice.
